# Venous blood parameters in determination of respiratory impairment in amyotrophic lateral sclerosis

**DOI:** 10.1038/s41598-023-42075-4

**Published:** 2023-09-21

**Authors:** Xianghua He, Jian Yang, Jiaming Feng, Hongyan Huang, Xiaolin Dong, Quanzhen Zhao, Qiuyan Shen, Caiyou Hu, Yanming Xu

**Affiliations:** 1 Department of Neurology, West China Hospital, Sichuan University, 37, Guo Xue Xiang, Chengdu, 610041 Sichuan China; 2 Department of Neurology, Jiangbin Hospital, No 85 Hedi Road, Guangxi Zhuang Autonomous Region, Nanning, 530021 China; 3 Department of Continuing Medical Education, West China Hospital, Sichuan University, 37, Guo Xue Xiang, Chengdu, 610041 Sichuan China; 4 Department of Clinical Medical College, West China Hospital, Sichuan University, 37, Guo Xue Xiang, Chengdu, 610041 Sichuan China; 5 Department of Neurology, The Affiliated Yan’An Hospital of Kunming Medical University, Kunming, 650051 Yunnan China

**Keywords:** Diseases of the nervous system, Motor control, Regeneration and repair in the nervous system, Sensorimotor processing

## Abstract

This study aimed to investigate the relationship between venous blood parameters and respiratory functions in patients with amyotrophic lateral sclerosis (ALS) and develop a model to predict respiratory impairment for individual patients with ALS. A total of 416 ALS patients were included in the study, and various hematologic and biochemical laboratory parameters as well as demographic and clinical factors were collected and compared. A multivariable logistic regression model was constructed to assess the association between FVC and venous blood biomarkers and clinical factors. The results showed that along with onset age, bulbar-onset, disease duration, BMI, eosinophil count (EO#), basophil count (BASO#), creatinine (CREA), uric acid (URCI) and low-density lipoprotein cholesterol/high-density lipoprotein cholesterol (LDL/HDL) ratio were associated with reduced FVC. The area under the ROC curve is 0.735 for the test set and 0.721 for the validation set. The study also developed a relatively acceptable model for predicting respiratory impairment in ALS patients. These findings suggest that EO#, BASO#, CREA, URIC and LDL/HDL ratio can be useful in assessing FVC in ALS and can be easily accessible, accurate, and low-cost parameters.

## Introduction

Amyotrophic lateral sclerosis (ALS) is a progressive neurodegenerative disorder with a variable natural history, which affects 0.6–3.8 per 100,000 person/year worldwide^1^. The initial clinical presentation and evolution of ALS is heterogeneous^2^. First symptoms usually occur in the fifth or sixth decade of life^1^. Most patients die within 3–5 years after onset, generally due to respiratory failure^3^. Thus, monitoring respiratory function is of significant importance for ALS patients^4, 5^.

Recently, several biochemical parameters have been demonstrated to be related to respiratory impairment^6–10^. EMPOWER study founded a weak longitudinal correlation between vital capacity and plasma creatinine^6^. Adriano Chiò et. al. suggested that lower albumin and creatinine (CREA), levels were strongly related to forced vital capacity (FVC)^7^. And cross-sectional study from Japan suggested that FVC was associated with serum levels of total cholesterol, low-density lipoprotein cholesterol (LDL), CREA, and urate^8^. Study from USA and Poland also showed that plasma CREA correlated with FVC^9, 10^. These studies suggested that there were some correlations between hematologic and biochemical laboratory parameters and respiratory function. However, the latest study did not confirm a correlation between plasma CREA and FVC^11^. The contradictory results suggested that further studies of blood biomarkers and FVC are needed.

The aim of the present study was to analyze a potential utility of readily available, relatively inexpensive, and rapid to determine laboratory parameters in the assessment of respiratory impairment in patients with ALS; and to develop and validate a model of hematologic and biochemical parameters for predicting respiratory impairment for patients with ALS.

## Methods

### Ethics statement

This study protocol was reviewed and approved by Ethics Committee of West China Hospital, Sichuan University, approval number 2020-842 and 2021-799, and all the participants signed written informed consent. All protocols and procedures of our research were conducted ethically in accordance with the World Medical Association Declaration of Helsinki. All methods were performed in accordance with the relevant guidelines and regulations.

### Patients

Patients from two cohorts were included in our study. In the retrospective cohort, 319 patients with ALS were enrolled from 1 January 2015 to 30 December 2020. And in the prospective cohort, 97 participants from 1 March 2017 to 30 October 2021 were enrolled. Our medical center was a large, regional referral ALS clinic located in Southwest China. The diagnosis of ALS was made according to the revised-El Escorial criteria for probable or definite ALS^12, 13^. Patients with other medical or neurological diseases and patients with missing baseline hematologic and biochemical values were excluded. Patients with manifestations of neoplastic disorders or patients who could not perform respiratory function were also excluded.

### Clinical variables

The clinical analysis included age, sex, age at ALS onset, disease duration, onset body region (limb/bulbar), diagnostic level (probable/definite), blood pressure and comorbidities. We extracted data on comorbidities present at diagnosis as listed in medical records. Charlson comorbidity index (CCI) was used to evaluate comorbid conditions^14^. Weight and height were measured following international guidelines, and body mass index (BMI) was calculated as weight (kg)/height (m)^2^ at the time of pulmonary function tests. High blood pressure was defined as systolic blood pressure (SBP) above 140 mmHg and/or diastolic blood pressure (DBP) above 90 mmHg in this study.

Venous blood parameters were obtained using an automated hematology analyzer (KX-21 N, Sysmex America, Lincolnshire, IL, USA). Hematologic data on platelet count (PLT), white blood count (WBC), neutrophil count (NEUT#), lymphocyte count (LYMPH#), monocyte count (MONO#), eosinophil count (EO#), and basophil count (BASO#) as well as and biochemical parameters on albumin (ALB), glucose (GLU), urea (UREA), creatinine (CREA), uric acid (URIC), triglyceride (TG), cholesterol (CHOL), low-density lipoprotein cholesterol (LDL), high-density lipoprotein cholesterol (HDL), and creatine kinase (CK) were obtained. PLR was calculated as the ratio of PLT to LYMPH# and NLR as the ratio of NEUT# to LYMPH#. LDL/HDL was measured as the ratio of LDL to HDL. FVC was measured using a standard volumetric spirometer, with the patient in a standing position. Spirometer was quality controlled using 2005 criteria^15^. The spirometry methods produce comparable measurements of forced vital capacity (FVC)^16^. At least three acceptable manoeuvres were performed. Obstructive physiology was defined as the presence of airflow limitation with FEV1/FVC < 0.70^17^. For participants without airflow limitation (FEV1/FVC ≥ 0.7), restrictive physiology was operationalized as FVC < 80% predicted^17^.

### Statistical analysis

(1) Correlation analysis: Continuous variables with normal distribution were reported as mean ± standard deviation; non-normal variables were presented as median (interquartile range [IQR]). The linear correlations of these variables and the FVC were calculated by Spearman. For categorical variables, the correlations were performed by the nonparametric test (Wilcoxon rank sum test). For normal distribution data, differences were performed by Independent Samples T-Test. For non-normal distribution, differences were assessed for significance using the Mann–Whitney test or chi-squared tests. (2) The training set and the test set: The retrospective cohort was divided into two independent sets by random sampling: a training set and a test set. The training set included 70% (220/319) of the retrospective cohort. Four participants with missing data were deleted. The test set included 30% (95/319) of the retrospective cohort. Models were developed using the characteristics in the training set. To verify the model, the test set was used to validate the models for internal validation, and the prospective cohort for external validation, respectively. In order to improve the robustness of the model, fivefold cross-validation was used. And down sampling method was used to deal with the class imbalance problem. (3) Logistic regression: Logistic regression was used to construct a model to predict FVC. For factors among each other (NEUT# vs. WBC, onset.age vs. age, LDL vs. CHOL) with rho > 0.75 in correlation analysis, only one factor was screened out in the next stage of the analysis. For groups of factors (weigh, height and BMI; NEUT#, LYMPH#, and PLR; PLT, LYMPH#, and NLR; LDL, HDL and LDL/HDL; SP, DP and hypertension) with strong clinical correlation, only BMI, PLR, NLR, LDL/DHL and hypertension were screened out for the next stage of analysis. Correlation analysis between each variable and FVC was carried out to determine factor for logistic regression. Then, a total of 23 variables including gender, disease duration, onset age, site of onset, diagnostic level, CCI, blood pressure category (hypertension or not), BMI, WBC, NLR, PLR, MONO#, EO#, BASO#, ALB, GLU, UREA, CREA, URIC, TG, CHOL, LDL/HDL, and CK were included to construct the model. Based on the results of backward stepwise regression method combined with Akaike information criterion (AIC), the equation of FVC for predicting respiratory impairment in ALS patients were established. Heatmap of FVC was depicted. Receiver operating characteristic (ROC) curves was used to assess the prediction accuracy of the model. All statistical analyses were performed using SPSS 24.0 (IBM, Chicago, IL, USA) and R 4.1.0 (www.rproject.org).

## Results

There were 92 male patients (69.70%) with FVC < 3.062 and 40 female patients (30.30%) with FVC < 2.266 in the retrospective cohort (Table 1). In the prospective cohort, there were a similar percentage of male (66.67%) and female (33.33%) ALS individuals with abnormal FVC (Table 1). There were significant differences in age, onset age, weight,

**Table 1:** Comparison of the analyzed clinical and laboratory data in ALS patients stratified by FVC.

Variable	The retrospective cohort				The prospective cohort			
	Total	FVC: normal	(Male and FVC < 3.062) or (female and FVC < 2.266)	P	Total	FVC: normal	(Male and FVC < 3.062) or (female and FVC < 2.266)	P
Gender	319	187	132		97	58	39	
Male	230	138 (73.80%)	92 (69.70%)	0.421	59	33 (56.90%)	26 (66.67%)	0.334
Female	89	49 (26.20%)	40 (30.30%)		38	25 (43.10%)	13 (33.33%)	
Age (year)	319	51.75 ± 9.72	59.71 ± 10.83	< 0.001	97	52.53 ± 11.00	61.64 ± 10.46	< 0.001
Onset age (year)	319	50.62 ± 9.77	58.35 ± 11.19	< 0.001	97	51.67 ± 10.95	60.44 ± 10.52	< 0.001
Hypertension	315	184	131		97	58	39	
SBP (mmHg)	315	130.00 (23.00)	130.00 (25.00)	0.387	97	125.00 (25.00)	132.00 (35.00)	0.319
DBP (mmHg)	315	88.00 (17.00)	84.00 (19.00)	0.022	97	81.43 ± 12.42	86.21 ± 14.02	0.486
Weight (kg)	319	63.00 (14.00)	55.00 (10.75)	< 0.001	97	60.19 ± 8.96	55.00 ± 8.87	0.006
Height (m)	319	1.63 ± 0.08	1.58 ± 0.07	< 0.001	97	1.61 ± 0.07	1.59 ± 0.08	0.110
BMI (kg/m^2^)	319	23.26 ± 3.02	21.81 ± 2.84	< 0.001	97	23.03 ± 2.57	21.79 ± 3.29	0.041
Site of onset	319	187	132		97	58	39	
Limb onset	259	159 (85.03%)	100 (75.76%)	0.092	75	46 (79.31%)	29 (74.36%)	0.568
Bulbar onset	51	23 (12.30%)	28 (21.21%)		22	12 (20.69%)	10 (25.64%)	
Other	9	5 (2.67%)	4 (3.03%)		0	0	0	
Diagnostic level	319	187	132		97	58	39	
Probable	155	94 (50.27%)	61 (46.21%)	0.475	70	44 (75.86%)	26 (66.67%)	0.322
Definite	164	93 (49.73%)	71 (53.79%)		27	14 (24.14%)	13 (33.33%)	
Disease duration (m)	319	11.00 (11.00)	12.00 (17.00)	0.072	97	11.50 (10.00)	12.00 (18.00)	0.205
PLT (10^9^/L)	319	183.00 (80.00)	183.00 (70.75)	0.815	97	168.00 (75.25)	186.00 (65.00)	0.079
WBC (10^9^/L)	319	5.83 (1.97)	5.83 (2.36)	0.412	97	5.31 (1.69)	5.36 (1.83)	0.965
NEUT# (10^9^/L)	319	3.39 (1.60)	3.41 (1.54)	0.826	97	3.21 ± 0.98	3.40 ± 1.14	0.401
LYMPH# (10^9^/L)	319	1.82 (0.58)	1.64 (0.79)	0.007	97	1.60 (0.82)	1.41 (0.84)	0.106
PLR	319	101.72 (51.15)	114.58 (67.12)	0.023	97	95.49 (63.58)	127.45 (84.11)	0.037
NLR	319	1.87 (1.10)	2.05 (1.00)	0.116	97	1.91 (0.95)	2.04 (1.45)	0.131
MONO# (10^9^/L)	319	0.41 (0.18)	0.39 (0.15)	0.595	97	0.38 (0.15)	0.39 (0.18)	0.962
EO# (10^9^/L)	318	0.12 (0.12)	0.11 (0.12)	0.296	97	0.15 (0.16)	0.12 (0.15)	0.306
BASO# (10^9^/L)	318	0.03 (0.02)	0.03 (0.02)	0.834	97	0.02 (0.02)	0.02 (0.01)	0.29
ALB (g/L)	319	42.70 (4.40)	42.20 (4.68)	0.144	97	42.15 (3.88)	41.90 (4.90)	0.991
GLU (mmol/L)	319	4.72 (0.83)	4.94 (0.69)	0.018	97	4.68 (0.70)	4.72 (0.84)	0.659
UREA (mmol/L)	319	5.00 (1.50)	5.30 (2.08)	0.154	97	5.50 (2.03)	5.50 (2.30)	0.664
CREA (µmol/L)	319	62.00 (16.00)	56.00 (19.00)	< 0.001	97	61.00 (19.25)	59.00 (33.00)	0.985
URIC (µmol/L)	319	311.00 (86.00)	303.50 (103.25)	0.449	97	291.00 (101.50)	280.00 (114.00)	0.749
TG (mmol/L)	319	1.33 (1.04)	1.15 (0.68)	0.004	97	1.32 (0.95)	1.12 (0.43)	0.050
CHOL (mmol/L)	319	4.60 ± 0.86	4.66 ± 0.90	0.566	97	4.42 ± 0.83	4.69 ± 1.08	0.165
CK (mmol/L)	319	165.00 (209.00)	170.50 (183.50)	0.984	97	143.00 (137.75)	104.00 (103.00)	0.109
LDL (mmol/L)	319	2.73 ± 0.73	2.69 ± 0.79	0.651	97	2.66 ± 0.69	2.83 ± 0.87	0.276
HDL (mmol/L)	319	1.19 (0.42)	1.34 (0.50)	< 0.001	97	1.21 (0.33)	1.31 (0.39)	0.025
LDL/HDL	319	2.38 (1.07)	2.08 (1.05)	0.001	97	2.19 (0.92)	1.90 (1.19)	0.199
CCI	319	0.00 (0.00)	0.00 (1.00)	0.056	97	0.00 (0.00)	0.00 (0.00)	0.690

*SBP* systolic blood pressure, *DBP* diastolic blood pressure, *BMI* body mass index, *PLT* platelet count, *WBC* white blood count, *NEUT#* neutrophil count, *LYMPH#* lymphocyte count, *PLR* the ratio of PLT to LYMPH#, *NLR* the ration of NEUT# to LYMPH#, *MONO#* monocyte count, *EO#* eosinophil count, *BASO#* basophil count, *ALB* albumin, *GLU* glucose, *UREA* urea, *CREA* creatinine, *URIC* uric acid, *TG* triglyceride, *CHOL* cholesterol, *CK* creatine kinase, *LDL* low-density lipoprotein cholesterol, *HDL* high-density lipoprotein cholesterol, *LDL/HDL* the ratio of LDL to HDL, *CCI* Charlson Comorbidity Index.

BMI, PLR, TG and HDL when stratified by FVC either in the retrospective cohort or in the prospective cohort (all P < 0.05, Table 1). Statistically significant correlations were observed between older age, later onset age, lower weight, lower BMI, lower levels of PLR and TG, and higher levels of HDL with reduced FVC.

**Figure 1:**
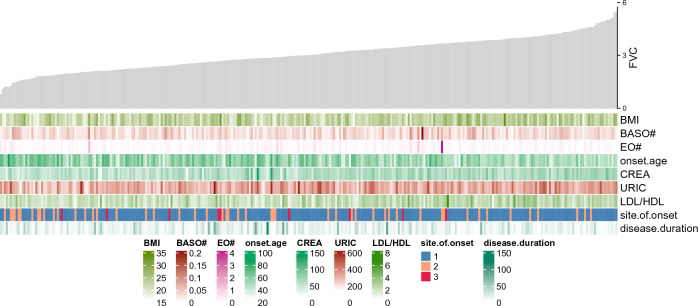
Heatmap of FVC in this study. The grey histogram represents values of FVC. Below the histogram are factors in the logistic regression model equation. The brightness of the color varied upon the value for continuous variables. *FVC* forced vital capacity, *BMI* body mass index, *BASO#* basophil count, *EO#* eosinophil count, *CREA* creatinine, *URIC* uric acid, *LDL* low-density lipoprotein cholesterol, *HDL* high-density lipoprotein cholesterol, *LDL/HDL* the ratio of LDL to HDL.

To further analyze the correlation between these factors and FVC, we developed a multivariable logistic regression model for the association between the hematologic, biochemical laboratory parameters, clinical factors and FVC in ALS patients in the test set. The regression equation was created from the Estimate values obtained by z-value and was presented in Table 2. Moreover, we constructed a heatmap to show the relation between these variables in the equation and FVC (Fig. 1).

**Table 2:** Variable in the prediction model by backward stepwise regression method combined with AIC for logistic regression equation.

Variable	Estimate	Std.Error	z.value	P-value
(Intercept)	0.022	1.439	0.015	0.988
BMI	0.199	0.053	3.770	0.000
EO#	1.029	0.621	1.658	0.097
BASO#	16.960	8.690	1.952	0.051
CREA	0.043	0.012	3.749	0.000
URIC	0.004	0.002	2.163	0.031
LDL/HDL	0.260	0.181	1.430	0.153
Disease.duration	0.021	0.009	2.220	0.026
Onset.age	0.101	0.016	6.218	0.000
Site.of.onset 2 (bulbar onset)	0.839	0.428	1.961	0.050

*BMI* body mass index, *EO#* eosinophil count, *BASO#* basophil count, *CREA* creatinine, *URIC* uric acid, *LDL* low-density lipoprotein cholesterol, *HDL* high-density lipoprotein cholesterol, *LDL/HDL* the ratio of LDL to HDL.

Model FVC = 0.022 + 0.199 BMI + 1.029 EO# + 16.960 BASO# + 0.043 CREA level + 0.004 URIC level + 0.260 LDL/HDL + 0.021 disease duration + 0.101 Onset.age + 0.839 Site.of.onset 2 (bulbar onset) (Table 2).

**Figure 2:**
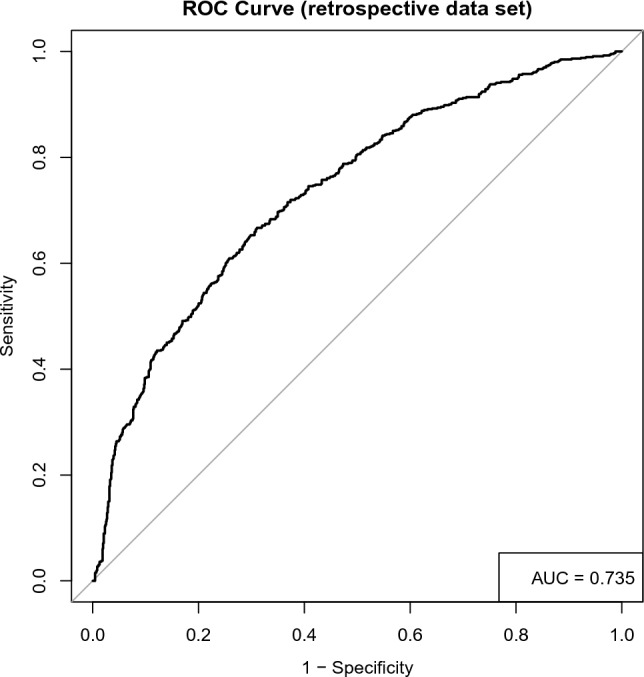
Receiver operating characteristic (ROC) curve for the backward stepwise regression method combined with AIC model for predicting FVC in ALS patients in the test set.

**Figure 3:**
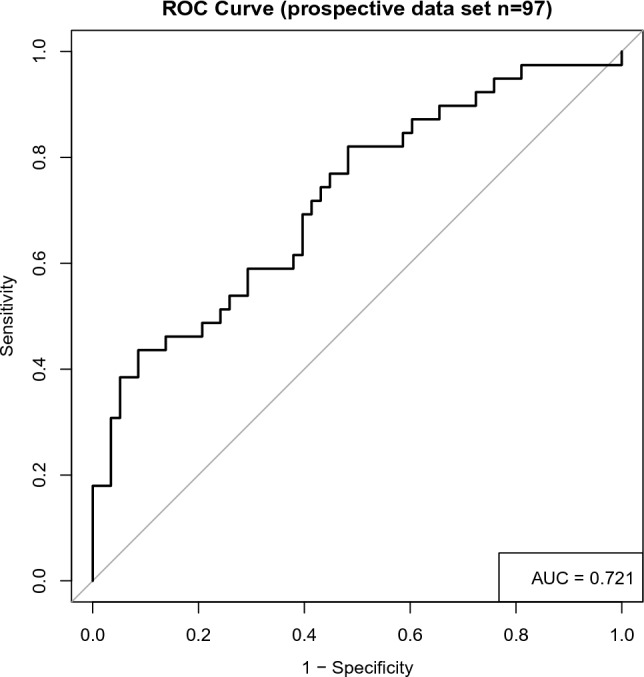
Receiver operating characteristic (ROC) curve of FVC in the validation set.

Receiver operating characteristic (ROC) curve with 9 predictive variables revealed that the area under the curve was 73.5% in the test set (Table 3, Fig. 2). And the ROC curve has standard error of 0.013 with 95% confidence interval as 0.710–0.760 in the test set. To validate the model, we tried to predict the data from the prospective cohort and calculated the ROC curve, which yielding a concordance statistic of 0.721 (95% CI 0.616–0.825) (Table 3, Fig. 3).

**Table 3:** ROC curve for the test set and the validation set.

	The test set	The validation set
Area under the ROC curve (AUC)	0.735	0.721
Standard error	0.013	0.053
95% Confidence interval	0.710–0.760	0.616–0.825
Z statistic	18.529	4.127
Significance level P (area = 0.5)	1.202E−76	3.679E−05

## Discussion

Respiratory failure is the main cause of death in ALS patients^3^. It is of significance to find some easily accessible, accurate, and low-cost parameters to assess the respiratory function. We created and validated a multivariable logistic regression model for the association between FVC and the venous blood biomarkers and clinical factors in ALS patients. Our single-site study found that FVC related to hematologic and biochemical laboratory parameters. EO#, BASO#, CREA, LDL /HDL and URIC are easily accessible, accurate, and low-cost parameters useful in assessment of the FVC in ALS.

To the best of our knowledge, there are few studies about models predicting respiratory function in ALS patients^11, 18^. In our model, onset age, site of onset, disease duration, BMI, CREA, LDL /HDL and URIC were factors previously reported to predict respiratory impairment^6–9, 18^. In addition, EO# and BASO# were two new features predicting to FVC in ALS. This prediction model could be useful in clinical settings in which the respiratory function is not available.

Older age and bulbar onset are consistently reported to have poorer outcomes^19^. BMI, onset age and disease duration were factors correlated with ALS prognostication either in retrospective or in prospective studies^20–23^. Recently, a large multinational study with participants aged 40 years and over founded that low BMI was one of the most influential risk factors for chronic airflow obstruction^24^, which implicated a potential reason for low BMI associated with impaired FVC in this study.

Lower level of CREA was related to impaired FVC in ALS in this study, which was consistent with previous studies^7–9^. In these studies, Ken Ikeda et.al found that the annual decline of FVC ≥ 30% was significantly linked to baseline serum levels of CREA^8^. Serum CREA is a product of nonenzymatic catabolism of creatine phosphate in muscles, and is transported from muscle through the circulation to the kidneys^25^.

A decreased level of plasma CREA in ALS patients is expected because of the variation in muscle mass observed in these patients. Moreover, CREA levels are correlated with lean body mass in healthy individuals^26^ and with BMI in ALS individuals^7^.

The mean TG level was significantly lower among patients with a lower FVC but not statistical significance at multivariable analysis. We confirmed that decreased LDL/HDL ratio was correlated with decreased FVC, as reported by previous studies^8, 18^. Whereas in a Dutch study, authors found that mean CHOL and LDL levels were lower in patients with FVC < 70%. Higher serum LDL/HDL ratio was correlated with increased survival^27^. Study from French reported that serum levels of CHOL and LDL were significantly increased in ALS patients, and the elevation of LDL /HDL ratio was associated with prolonged survival^28^. Ethical and environmental backgrounds may lead to different lipid levels in patients with ALS. Multicenter, prospective study are needed to elucidate the relationship between lipid and respiratory function.

Study from Japan showed that the rapid worsening of annual FVC was associated with serum levels of URIC^8^. Interestingly, the level of URIC was not statistically significant between FVC normal group and FVC lower group but independently related to decreased FVC at multivariable analysis in our study. Study from USA found strong hazard ratios relating plasma CREA and ALSFRS-R when using trajectories of all three measures of plasma CREA, plasm URIC, and ALSFRS-R to predict time to death. These studies suggest complex biochemical interactions exist in ALS patients. More studies are warranted to better understand the metabolic mechanisms of disease progression in ALS.

Peripheral EO# is an important clinical biomarker in the management of asthma and chronic obstructive pulmonary disease (COPD)^29, 30^. For persistent childhood asthma patients, normal lung function and serum EO# at baseline are clinical prognostic indicators of remission by adulthood^31^. For COPD patients, counts of 4% or greater or 300 cells per μL or more might identify a deleterious effect of inhaled corticosteroids withdrawal^32^. Furthermore, retrospective cohort study also shown that ALS patients concomitant with COPD relates to poor outcome^23^. One possibility of EO# predicting respiratory in this study is that some ALS patients coexisting with COPD, the other possibility is that ALS patient concomitant with some indirect factors which correlates with EO# and FVC. Further studies are warranted to understand the mechanism underpinning the association of EO# and FVC in ALS.

In respiratory disease, BASO# plays a distinct role in the pathogenesis of allergic and nonallergic^33^. Recently, authors found that the number of circulating BASO# was significantly elevated in patients with aspirin-exacerbated respiratory disease^34^. Except for respiratory disease, Katie Lunnon et al. have found increased numbers of BASO# in people with MCI and AD, Yet the basophil counts are within the normal acceptable range^35^. More studies are needed to clarify the mechanisms of BASO# and respiratory impairment in ALS and other neurodegenerative disorders.

Our results should be interpreted with caution and a number of limitations should be borne in mind. Firstly, the prediction model derived from the retrospective cohort could not analyze data potentially important to risk of FVC. For example, eosinophils and uric acid values measured at admission may be affected by the other conditions coexisted with ALS, which we did not analyze. Secondly, possible ALS patients were excluded in this study, thus the prediction model is only suitable for probable and definite ALS patients. Thirdly, the model should be tested on multicenter, prospective cohorts to study the validity and predictability.

In conclusion, our study indicates that EO#, BASO#, CREA, LDL/HDL and URIC might be important ALS biomarkers and may be used to assess FVC in ALS patients when lung function was not available. This finding needs to be validated in prospective, multi-site studies.

## Data Availability

All data generated or analysed during this study are included in this published article. The datasets used and/or analysed during the current study available from the corresponding author on reasonable request.
